# Molecules and Atoms

**Published:** 1887-05

**Authors:** 


### MOLECULES AND ATOMS.

A molecule is the smallest patricle of matter made up of a single combination of atoms ; the combining unit of a substance composed of atoms. The character of a molecule may be better understood by taking a grain of sand as a substance, crush it to a fine powder and *assume* the particles to be molecules ; these, in turn, are composed of atoms which united in certain proportions form the molecules. A single combination of atoms therefore into one mass, in which the affinities or chemical attractions of the atoms are satisfied, is called a molecule. Unlike the atom, the molecule may be disorganized and its atoms rebuilt into other molecules. *Elementary molecules* are those which are composed of atoms of the same elementary substance, and unite with other atoms in pairs, etc., as molecules instead of atoms, *integral molecules* are made up of atoms of different elementary substances.

All compound bodies are made up of molecules which are composed of atoms of elementary substances.

In conclusion, therefore, it may be stated that:

An *atom* is a chemical unit incapable of division.

A *molecule* is a physical unit, made up of and capable of being resolved into its component atoms.

An *atom* is the indivisible unit of an elementary substance. It is the smallest particle of an element that is known to exist. It is invisible, individual, indestructible, unchangeable, eternal. Science offers no proof that atoms have been or can be created or destroyed. It may therefore be assumed that atoms are primeval with time and co-existent with eternity; the only material substance in which there is no change. They may unite or associate themselves with other atoms to form *molecules*, but in whatever combination or relation they may exist they can be dissociated and restored to their original atomic unity.

The term *atom* can never be applied except to elementary substances, and we wish our readers to clearly understand the chemical meaning and signification of the word, for upon atoms and their relation to each other is built the Chemical Science of to-day.

